# PSYCHIATRIC COMORBIDITY IN A SAMPLE OF PATIENTS WITH COGNITIVE-BEHAVIORAL MINORITY DISEASE

**DOI:** 10.1192/j.eurpsy.2023.1560

**Published:** 2023-07-19

**Authors:** R. De la Mata, C. Manso-Bazús, S. Pujol, L. Torrent, L. Urraca, D. Vázquez-Tarrio, M. Esteve, E. Fernández, M. Pàmias

**Affiliations:** 1 Children and adolescescents Mental Health Service. Parc Taulí Hospital Universitari. Universitat Autònoma de Barcelona. Sabadell. Spain. CIBERSAM; 2 Center of Genomic Medicine, Hospital Parc Taulí, Sabadell; 3 ETSIAAB. Universidad Politécnica de Madrid, Universidad Politécnica de Madrid, Madrid, Spain; 4 Fundación Orienta, Barcelona

## Abstract

**Introduction:**

About the term cognitive-behavioral minority disease or rare disease are a group of diseases that affect between 6-8% of the populatio. It is estimated that there are more than 7000 in the world, the majority with a genetic basis and affect various organs and systems, they also present psychiactric comorbidities and cause a physical or mental disability. Given its definition, it is difficult to see a large number of these patients in our usual clinical activity, so their management can be complicated.

**Objectives:**

To evaluate the prevalence of psychiatric comorbidity and the prevalence of psyhcopharmacological treatment in children and adolescents whe present a minority disease.

**Methods:**

This is a descriptive, controlled, retrospective cross-sectional study of a sample obtained by non-probabilistic sampling, which is representative of the study population.

The statistical analysis was made using the statistical program SPSS V22 (2013).

**Results:**

With a sample of 114 patients, of which 26,6% presented fragile X syndrome, secondly 25,3% presented Prader-Willi Syndrome and 48,1% other chromosomal abnormalities.

By subgroups (male:female): in Prader-Willi syndrome 6:14 (30%:70%), in Fragile X syndrome 12:9 (57,14%: 42,86%) and in other diseases 25:13 (75,69%: 34,21%).

**Conclusions:**

The creation of clinical expert units makes the possibility to increase knowledge of diseases whose prevalence in the population, thanks to technological advances, is increasing and where scientific knowledge is still limited.

These units are also important, in order to be able to offer personalized intensive treatments in order to reduce polypharmacy. There is not a great difference between the minority diagnosis and polypharmacy, although there is less polypharmacy than expected, which may be the result of the success of the most intensive and personal psychotherapeutic intervention in the unit.

**Disclosure of Interest:**

None Declared

